# Quality wines in Italy and France: A dataset of protected designation of origin specifications

**DOI:** 10.1016/j.dib.2024.110408

**Published:** 2024-04-16

**Authors:** Sebastian Candiago, Simon Tscholl, Leonardo Bassani, Helder Fraga, Lukas Egarter Vigl

**Affiliations:** a Institute for Alpine Environment, Eurac Research, Viale Druso 1, Bolzano/Bozen 39100, Italy; b Department of Economics, Ca’ Foscari University of Venice, S. Giobbe 873, Venezia 30121, Italy; c Professorship of Ecological Services, Bayreuth Center of Ecology and Environmental Research (BayCEER), University of Bayreuth, Universitätsstraße 30, Bayreuth 95447, Germany; d Department of Ecology, University of Innsbruck, Innrain 52, Innsbruck 6020, Austria; e Centre for the Research and Technology of Agro-Environmental and Biological Sciences, University of Trás-os-Montes and Alto Douro, Vila Real 5000-801, Portugal

**Keywords:** Geographical indication, Categories of wine product, European Union, PDO, Wine quality

## Abstract

Italy and France are historically among the countries that produce the most prestigious wines worldwide. In Europe, these two countries together produce more than half of the wines classified under the Protected Designation of Origin (PDO) label, the strictest quality mark of food and wines in the European Union. Due to their long tradition in wine protection, Italy and France include highly detailed specifications in their wine PDO regulatory documents that are usually not available for other countries, such as specific information about the main cultivars that must be used to make each wine or the required planting density in the vineyards. However, this information is scattered throughout the documents of each wine production area and has never been extracted and homogenised in a unique dataset. Here, we present the first dataset that characterizes the PDO wines produced in Italy and France at very high detail based on the information from the official EU geographical indication register. For each country it includes a standardized list of the PDO wine names, linked with their specific requirements, such as the wine colour, type, cultivars used and maximum allowed yields. The unprecedented level of detail of this dataset allows for the first time the analysis of more than 5000 traditional wines and their legal and agronomic specifications. This gives insights into the interplay between the European Union quality regulation policy, the wine sector, and agronomic practices, enabling researchers and practitioners to analyze wine production in the context of specific regulations or economic scenarios.

Specifications Table

**Table:** 

Subject	Agricultural Economics; Agronomy and Crop Science; Management, Monitoring, Policy and Law
Specific subject area	Characterization of PDO wine products
Type of data	Table
Data collection	Extraction and standardization of information from the legal documents that regulate wine PDOs in Italy and France was carried out using the EU geographical indication register eAmbrosia as the main source. Where necessary, integration of missing data was done with information from the Ministry of Agriculture, Food Sovereignty and Forestry (for Italy) and the National Institute of Origin and Quality (for France).
Data source location	The data was collected from the official indication register eAmbrosia, the repository of the geographical indications for agri-food production, wine and spirits registered and protected in the EU, or from relevant national websites.
Data accessibility	Repository name: figshare Data identification number: 10.6084/m9.figshare.25393261.v2 Direct URL to data: https://figshare.com/articles/dataset/Quality_wines_in_Italy_and_France_a_dataset_of_protected_designation_of_origin_specifications/25393261

## 1 Value of the Data

• This dataset presents the first detailed database of protected designation of origin (PDO) wines produced in Italy and France, the two countries with the highest number of wine geographical indications in the European Union.
• The data allows to characterize and classify more than 5000 Italian and French PDO wines based on a set of selected regulatory specifications, providing a reliable data basis for research about key economic sectors in these countries.
• The dataset enables researchers and practitioners to analyze wine production in the context of specific regulations, socio-economic scenarios or agroecological conditions to improve our understanding of how the EU quality policy shapes the future of the wine sector.
• Coupled with the available geospatial data on wine protected designation of origin areas [1], the information included in this dataset allows their spatialization and therefore the study of the influence of major global drivers of change on high-quality wines, e.g., climate change and land-use/land -cover change.

## 2 Background

The Protected Designation of Origin (PDO) label protects European Union (EU) products including the strictest rules on name, production practices and origin [2]. Wines play a major role in the EU list of PDO products, accounting for more than 40% of all PDO products [3]. To be labelled as a PDO product, a wine needs to be formally recognized by the EU, which requires applicants to establish a direct link between its quality attributes and geographical origin in a regulatory document, that is then published online. The acknowledgment of the PDO status ensures multiple benefits, such as the protection of intellectual property rights, improved visibility, and access to new markets and funds [4].

Despite an effort toward the standardization of the regulatory documents, there are still many differences in the way information of PDO products is presented for each country [1]. Italy and France, due to the longer tradition in GI protection, include more detailed information in their regulatory documents compared to other countries [5]. Given the increasing importance of traditional winegrowing in these countries for export and global trade, it is essential to standardize this knowledge [6]. This will support research and policy making in the fields of quality protection, wine economics and law, improving the knowledge on European vineyard landscapes, which is especially important considering the potential impacts of global changes [7].

## 3 Data Description

**Fig. 1:**
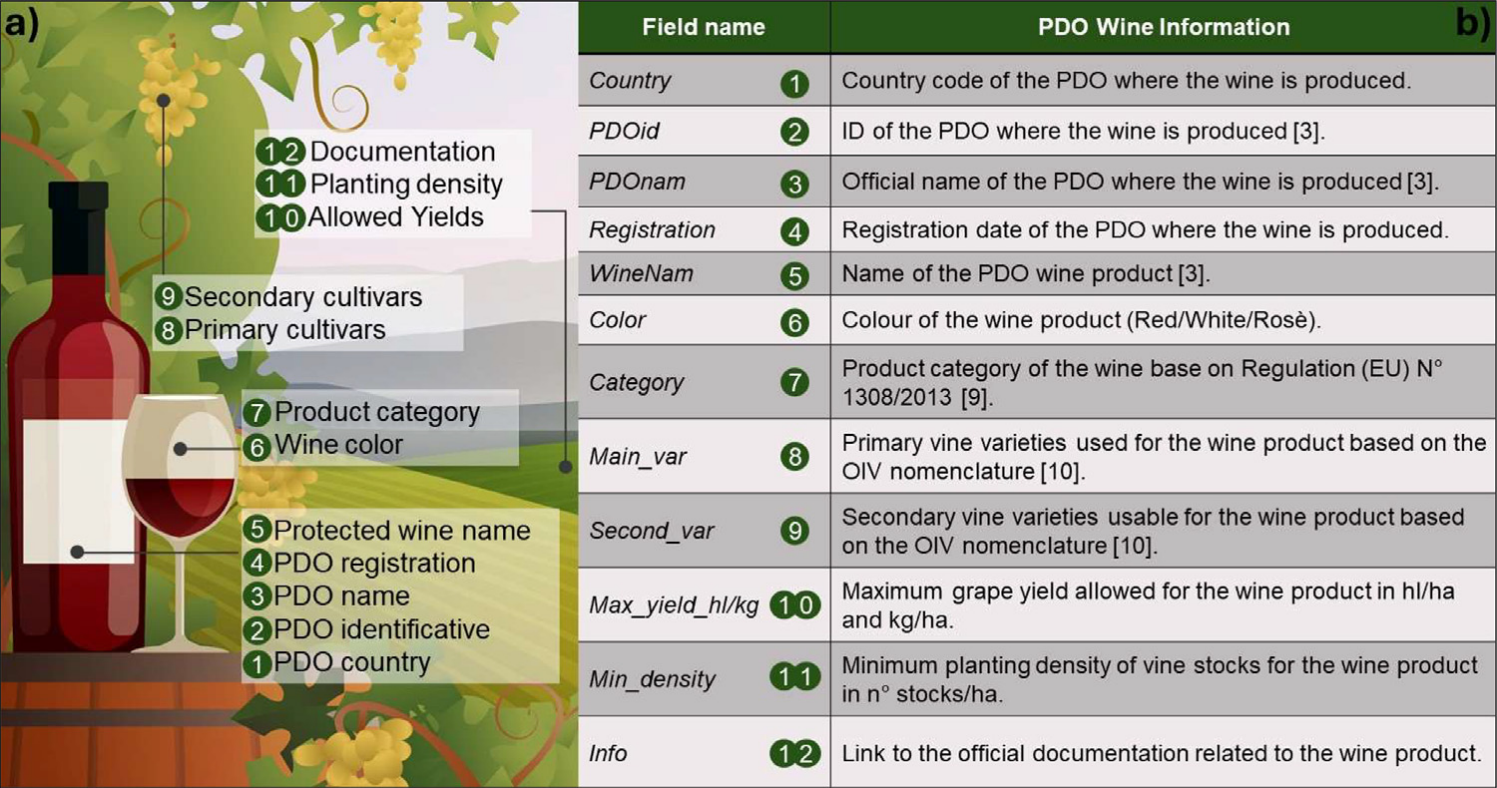
(a) PDO wine information included in our database (b) names of the fields included in our database and methodology for standardization. Each row corresponds to a unique field in the .csv dataset [9], [10], [11].

Our dataset comprises a set of regulatory information for all the PDO wines produced in Italy and France as of 04.11.2021, usable by researchers and decision makers (see Fig. 1). We collected only information that was standardizable among the two countries and the PDO wines. The information collected covers several specifications related to wine products protected by the PDO quality scheme. This includes the name of the wine product as well as information about the PDO where it is produced (e.g., name and registration date), the colour of the wine and the category of the wine product. The dataset also includes information on several agronomic practices that must be used when producing the wine, such as the main varieties that make up a wine and the additional varieties that may be added to the product in lower percentages. The varieties are spelled using the OIV nomenclature, the letter at the end of the variety commonly specifies the grape colour (e.g., N = noir, B = blanc, G = gris). Additionally, the collected data also specifies the minimum usable planting density and maximum yield from the vineyards for each wine product.

**Fig. 2:**
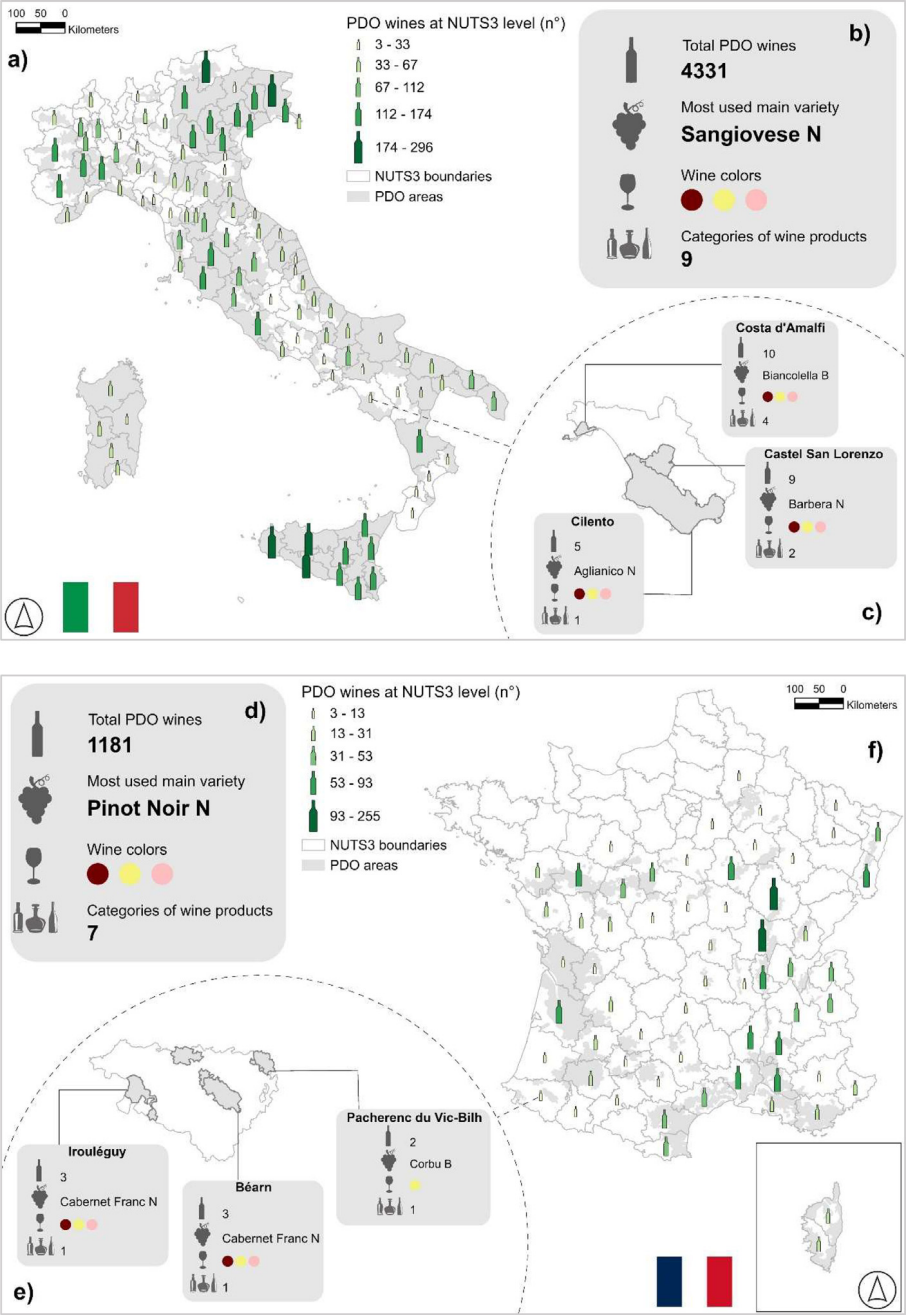
a and f, number of PDO wine products allowed in Italy and France (boundaries represent level 3 of the nomenclature of territorial units for statistics [12]); b and d, summary of some specifications included in the database for Italy and France; c and e, example of the characterization of different PDO areas based on the collected regulatory information. The variety name indicates the main variety that is used in the highest number of wine products from the respective area; if more than one main variety was used for an equal number of products, we only indicated the first one in alphabetical order. Grey areas represent the spatial extension of PDO areas in the two countries, PDO boundaries are taken from [1], icons are taken from [13].

This wine information can be easily combined with freely available data on the geographical boundaries of European PDO regions to gain more insights into the areas of production of PDO wines and to deepen their study by including a multiplicity of potential geospatial variables (see Fig. 2) [1]. The data is freely available through the Figshare data publisher [8] and it is provided in the form of a .csv file using UTF-8 encoding.

## 4 Experimental Design, Materials and Methods

**Fig. 3:**
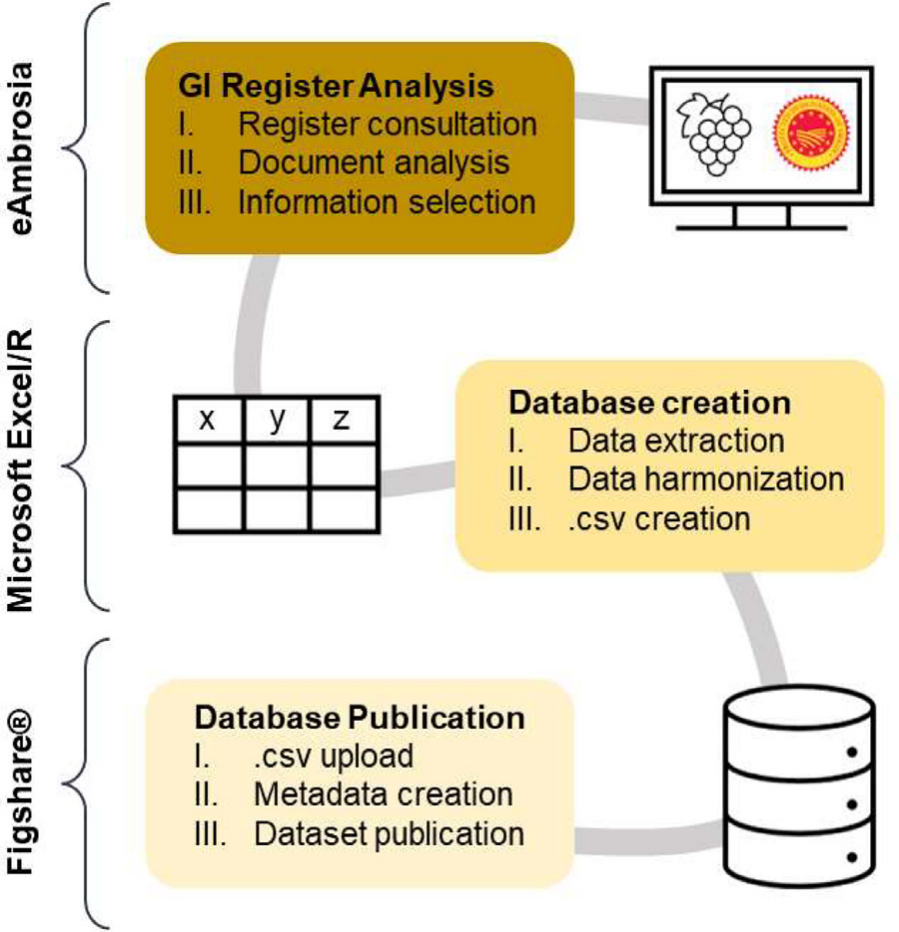
Conceptual representation of the experimental design, including the steps and tools used to create the database.

We created a tailored workflow to extract information from the official documents in the eAmbrosia portal for the PDO wine products of Italy and France [3]. The experimental design is outlined in Fig. 3.

To extract the regulatory information from the legal documents and insert them into our dataset, we consulted and manually downloaded the product specifications and other relevant regulatory documents of each PDO area. After analysing these documents, we selected a set of information to be extracted. We only collected regulatory information related to PDO wines that was available in PDO areas of both countries and that could be standardized among them. Next, we collected the relevant entries by copy-pasting them in a dedicated spreadsheet table before proceeding with their standardization using the Excel software [14]. Team members were fluent in Italian and French. The knowledge of the language was necessary because in many cases the documents are available only in the original language of the country of the PDO wine. If one of the documents was not included in the EU register, we looked for it in the national database from the Ministry of Agriculture, Food Sovereignty and Forestry (for Italy) or the National Institute of Origin and Quality (for France) [15,16].

## Limitations

Limitations related to the data collection include:

(i) the selection of information to be included in the dataset. The selected information was only extracted if it was comparable among the PDO products of both countries and thus could be standardized. Hence, certain parameters, e.g., some analytical or organoleptic characteristics of wines which are only available in a few special cases, were not included in the dataset;
(ii) possible transcription errors during the collection of the selected information from the official register of geographical indications eAmbrosia. To minimize errors in data gathering, random checks were conducted at various steps during the collection of the information;
(iii) amendments and future changes to the specifications of wine products. We present a comprehensive analysis as of 04.11.2021, however subsequent and future amendments might change the rules applicable to wine products of the PDO under analysis. These modifications could be tracked in the future by developing an automated tool to extract relevant information.

## Ethics Statement

The authors have read and followed the ethical requirements for publication in Data in Brief and confirm that the current work did not involve human subjects, animal experiments, or any data collected from social media platforms.

## CRediT authorship contribution statement

**Sebastian Candiago:** Conceptualization, Methodology, Validation, Formal analysis, Data curation, Writing – original draft, Writing – review & editing. **Simon Tscholl:** Conceptualization, Methodology, Validation, Formal analysis, Data curation, Writing – original draft, Writing – review & editing. **Leonardo Bassani:** Methodology, Formal analysis, Data curation. **Helder Fraga:** Methodology, Writing – review & editing. **Lukas Egarter Vigl:** Supervision, Methodology, Writing – review & editing.

## Data Availability

[15:47] Candiago, Sebastian (Guest) Quality wines in Italy and France: a dataset of protected designation of origin specifications (Original data) (figshare).
